# Theoretical and Experimental Analysis of Osmotically Assisted Reverse Osmosis for Minimum Liquid Discharge

**DOI:** 10.3390/membranes13100814

**Published:** 2023-09-27

**Authors:** Jaehyun Ju, Seoyeon Lee, Yusik Kim, Hyeongrak Cho, Sangho Lee

**Affiliations:** 1 School of Civil and Environmental Engineering, Kookmin University, 77 Jeongneung-ro, Seongbuk-gu, Seoul 02707, Republic of Korea; jhju@ktl.re.kr (J.J.); yeon623@kookmin.ac.kr (S.L.); yskim920930@kookmin.ac.kr (Y.K.); rhino@kookmin.ac.kr (H.C.); 2 Korea Testing Laboratory, 10, Chungui-ro, Jinju-si 52852, Republic of Korea; 3 Water Technologies Innovation Institute and Research Advancement (WTIIRA), Saline Water Conversion Corporation (SWCC), WQ36+XJP, Al Jubayl 35417, Saudi Arabia

**Keywords:** desalination, brine, minimum liquid discharge, membrane, osmotically assisted reverse osmosis, modeling, efficiency

## Abstract

Osmotically assisted reverse osmosis (OARO) is an innovative process that shows promising potential in the treatment of brine produced by conventional reverse osmosis (RO) systems. This study presents a theoretical and experimental analysis of the OARO process, focusing on its application to achieve minimum liquid discharge (MLD). This theoretical analysis includes the development of a mathematical model to describe the transport phenomena occurring during OARO. By considering mass balance equations coupled with transport equations, the theoretical model allows for the simulation of a full-scale system consisting of a single-stage RO and a four-stage OARO. Experimental investigations are also conducted to validate the theoretical model and to evaluate the performance of the OARO process. A laboratory-scale OARO system is designed and operated using a synthetic RO brine. Various operating conditions, including applied pressure, feed concentration, and draw concentration, are varied to investigate their effects on process performance. The experimental results demonstrate the feasibility of OARO as an MLD solution and also validate the predictions of the theoretical model, confirming its reliability for process optimization and design. The results of the theoretical analysis show that OARO has the potential to significantly improve water recovery compared to conventional RO. Based on the simulation, the optimal operating conditions are explored, leading to a significant reduction (up to 89%) in the volume of brine discharge.

## 1. Introduction

Water scarcity and environmental sustainability are pressing challenges in today’s world [1]. The need for efficient water treatment and recycling methods has never been more critical [2,3]. Meeting these challenges requires a multifaceted approach that combines technological innovation, policy reform, and public awareness [4,5]. Alternative water resources such as seawater desalination and wastewater reclamation can play a key role in increasing water supplies [6,7,8]. Reverse osmosis (RO) has been a cornerstone in providing these alternative water resources [9,10,11]. However, traditional RO systems often face problems in the form of brine, a concentrated salt solution produced as a by-product of the RO process [12,13]. This brine contains the impurities and salts removed from the water as well as additional chemicals used during the treatment process [14]. The disposal of RO brine can have a negative impact on the environment [15]. If not properly managed, brine discharge into natural water bodies can disrupt aquatic ecosystems by increasing salinity levels and harming aquatic life [15].

In this context, the concept of minimum liquid discharge (MLD) has emerged as an innovative water management approach aimed at minimizing the generation of waste brine in industrial processes, particularly in the context of desalination and wastewater reuse [16,17]. In contrast to traditional methods, MLD seeks to recover as much water as possible from the treated feedwater, offering several advantages such as reduced environmental impact, additional water and resource recovery, and regulatory compliance [18]. Nevertheless, the implementation of MLD requires specialized technologies that can “squeeze” water from the brine [19]. Traditional RO cannot be used because the pressure required for brine treatment is too high and may exceed the maximum allowable pressure of RO membranes (>90 bar) [20]. Although thermal processes such as multi-effect distillation (MED) and mechanical vapor compression (MVC) can be used for brine treatment, they are expensive and energy-intensive [21]. These challenges led to the exploration of alternative methods, such as osmotic-assisted reverse osmosis [22].

OARO is a hybrid membrane process that combines the principles of forward osmosis (FO) and RO [23]. By utilizing a draw solution to create an osmotic pressure gradient, OARO can overcome some of the limitations of traditional RO [24]. This osmotic gradient assists in water transport across the membrane, reducing the required hydraulic pressure and potentially lowering energy consumption [25]. OARO’s ability to achieve higher water recovery rates makes it a promising technology for MLD applications [26]. OARO also offers a higher energy efficiency than MED or MVC [27]. Due to its potential, OARO was recently investigated in previous works [22,23,24,25,26,27,28]. Nevertheless, OARO technology is in its early stages, and, thus, the stability and reliability of its process efficiency have not yet been fully verified. Moreover, membrane fouling may occur to reduce the process efficiency. Unfortunately, insufficient information is available on the understanding and optimization of OARO for MLD approaches in seawater desalination.

This paper provides an experimental and theoretical approach to investigate the potential and performance of OARO in the context of MLD for seawater desalination. It explores the implementation of lab-scale experiments, the development of performance prediction models, and the simulation of a full-scale OARO system, providing insights into the future prospects of this emerging technology. To the best of the authors’ knowledge, a systematic approach using theoretical and experimental methodologies to analyze the efficiency of both lab-scale and full-scale OARO systems has not been previously investigated.

## 2. Theory

### 2.1. OARO and Related Membrane Processes

There are several osmotic membrane processes, including pressure-assisted forward osmosis (PAFO), forward osmosis (FO), pressure-retarded osmosis (PRO), osmotically assisted RO (OARO), and reverse osmosis (RO). The general equation describing water transport in these processes is [24,29]

(1) $$J_{w}=A\left(P_{H}-P_{L}-\left(\pi_{H}-\pi_{L}\right)\right)=A\left(\Delta P-\Delta \pi \right)$$

where *J_w_* is the water flux (L/m^2^-hr), *A* is the water permeability of the membrane (L/m^2^-hr-bar), *P_H_* is the pressure on the high-salinity solution side, *P_L_* is the pressure on the low-salinity solution side, Δ*P* is the transmembrane pressure between the high-salinity and low-salinity solutions, and Δ*π* is the transmembrane osmotic pressure between the high-salinity and low-salinity solutions.

**Figure 1:**
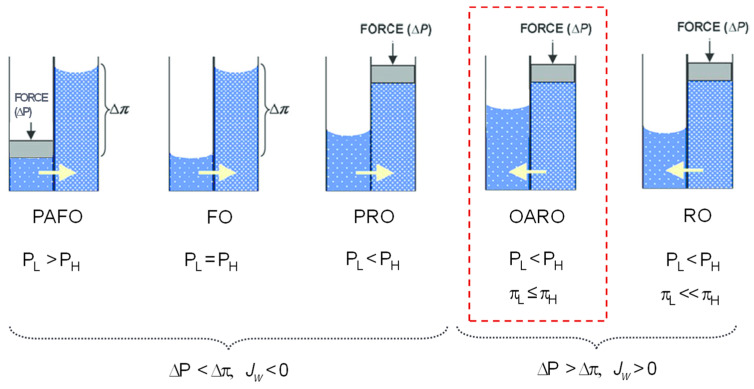
Comparison of flux directions and driving forces in various osmotic membrane processes, including pressure-assisted forward osmosis (PAFO), forward osmosis (FO), pressure-retarded osmosis (PRO), osmotically assisted RO (OARO), and reverse osmosis (RO).

Depending on the relative magnitudes of the applied and osmotic pressures, the characteristics of the membrane processes are determined. For instance, Δ*P* is positive and higher than Δ*π* in RO, PRO, and OARO. On the other hand, Δ*P* is zero in FO and negative in PAFO. The permeate flux in RO is driven by only the hydraulic pressure but that in OARO is driven by both the hydraulic and osmotic pressure. Accordingly, Δ*π* is higher for RO than for OARO, suggesting that the Δ*P* required for the process operation is lower for OARO than for RO (Figure 1).

### 2.2. Flux Equations for OARO

Similar to PRO and PAFO, a modified solution–diffusion model can be used to calculate the water flux and the salt fluxes [24,29]:

(2) $$J_{w}=A\left(\frac{\pi_{D}e^{-J_{w}k_{D}}-\pi_{F}e^{\frac{J_{w}}{k_{F}}}}{1-\frac{B}{J_{w}}\left(e^{-J_{w}k_{D}}-e^{\frac{J_{w}}{k_{F}}}\right)}+\Delta P\right)$$

(3) $$J_{s}=B\left(\frac{c_{F}e^{\frac{J_{w}}{k_{F}}}-c_{D}e^{-J_{w}k_{D}}}{1-\frac{B}{J_{w}}\left(e^{-J_{w}k_{D}}-e^{\frac{J_{w}}{k_{F}}}\right)}\right)$$

where *J_w_* is the water flux (L/m^2^-hr), *J_s_* is the salt flux (mole/m^2^-hr), *A* is the water permeability of the membrane (L/m^2^-hr-bar), *B* is the salt permeability of the membrane (L/m^2^-hr), *c_F_* is the salt concentration of the feed solution (mole/L), *c_D_* is the salt concentration of the draw solution (mole/L), *π_F_* is the osmotic pressure of the feed solution (bar), *π_D_* is the osmotic pressure of the draw solution (bar), *k_F_* is the mass-transfer coefficient related to the external concentration polarization (L/m^2^-hr), and *k_D_* is the mass-transfer resistance related to the internal concentration polarization (m^2^-hr/L). *k_F_* and *k_D_* can be calculated using the following equations [30]:

(4) $$k_{D}=\frac{S}{D}$$

(5) $$k_{F}=\frac{ShD}{d_{h}}=1.85\left(\frac{D}{d_{h}}\right)\left(\frac{u_{h}d_{h}}{\nu }\right)\left(\frac{\nu }{D}\right)\left(\frac{d_{h}}{L_{h}}\right)=1.85\left(\frac{u_{h}d_{h}}{L_{h}}\right)$$

where *S* is the structural parameter of the membrane (m), *D* is the diffusion coefficient of the solute (m^2^/s), *Sh* is the Sherwood number, *d_h_* is the hydraulic diameter (m), *L_h_* is the length of the membrane channel (m), *u_h_* is the crossflow velocity (m/s), and ν is the kinematic viscosity (m^2^/s).

As can be seen in the equations above, there are factors that affect the flux of OARO, including flow rate, pressure, concentration polarization, and fouling. With an increase in the flow rate, the external concentration polarization is suppressed, resulting in an increase in the flux. As the applied pressure increases, the flux generally increases. However, it also increases both the internal and external concentration polarization, which also affects the flux. When fouling occurs, the water permeability (*A*) of the membrane decreases, resulting in a decrease in the flux. Sometimes fouling also changes the salt permeability (*B*), which can affect the flux.

### 2.3. Mass Balance Equations for Full-Scale OARO Systems

**Figure 2:**
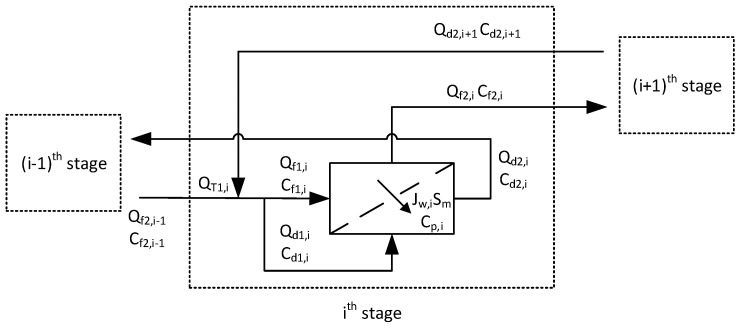
Flow diagrams for the ith stage in OARO process.

To simulate a full-scale OARO system, it is necessary to consider mass balance equations for water and the salt. In fact, it consists of several stages, and the mass balance equations should be solved in each stage [23]. Figure 2 shows flow diagrams for the *i*th stage in a OARO process. First, the total inflow to the stage is split into two streams, including the feed and draw flows. This gives the following equations:

(6) $$Q_{T,i}=Q_{f1,i}+Q_{d1,i}$$

(7) $$c_{T,i}Q_{T,i}=c_{f1,i}Q_{f1,i}+c_{d1,i}Q_{d1,i}$$

where *Q_T,i_* is the flow rate of the total inflow in the *i*th stage (m^3^/hr), *Q_f_*_1,*i*_ is the flow rate of the solution supplied to the feed side of the membrane in the *i*th stage (m^3^/hr), *Q_d_*_1,*i*_ is the flow rate of the solution supplied to the draw side of the membrane in the *i*th stage (m^3^/hr), *c_T,i_* is the concentration of the total inflow in the *i*th stage (g/L), *c_f_*_1,*i*_ is the concentration of the solution supplied to the feed side of the membrane in the *i*th stage (g/L), and *c_d_*_1,*i*_ is the concentration of the solution supplied to the draw side of the membrane in the *i*th stage (g/L). Here, the ratio of the feed to the total inflow (*FR_i_*) is defined by

(8) $$FR_{i}=\frac{Q_{f1,i}}{Q_{T,i}}=\frac{Q_{f1,i}}{Q_{f1,i}+Q_{d1,i}}$$

When the feed and effluent streams are fed to a membrane module in a countdown fashion, the feed is concentrated, and the effluent is diluted. Again, the following equations are obtained:

(9) $$Q_{f1,i}+Q_{d1,i}=Q_{f2,i}+Q_{d2,i}$$

(10) $$c_{f1,i}Q_{f1,i}+c_{d1,i}Q_{d1,i}=c_{f2,i}Q_{f2,i}+c_{d2,i}Q_{d2,i}$$

where *Q_f_*_2,*i*_ is the flow rate of the concentrated solution from the feed side of the membrane in the *i*th stage (m^3^/hr), *Q_d_*_2,*i*_ is the flow rate of the diluted solution from the draw side of the membrane in the *i*th stage (m^3^/hr), *c_f_*_2,*i*_ is the concentration of the concentrated solution from the feed side of the membrane in the *i*th stage (g/L), and *c_d_*_2,*i*_ is the concentration of the diluted solution from the draw side of the membrane in the *i*th stage (g/L).

A portion of the feed passes through the membrane to be mixed with the effluent. If the rejection of the membrane is not 100%, a small fraction of the salt also passes from the feed to the draw. These can be described by the following equations:

(11) $$Q_{f1,i}-J_{w,i}S_{m,i}=Q_{f2,i}$$

(12) $$c_{f1,i}Q_{f1,i}-c_{p,i}J_{w}{}_{,i}S_{m,i}=c_{f2,i}Q_{f2,i}$$

(13) $$Q_{d1,i}+J_{w,i}S_{m,i}=Q_{d2,i}$$

(14) $$c_{d1,i}Q_{d1,i}+c_{p,i}J_{w,i}S_{m,i}=c_{d2,i}Q_{d2,i}$$

where *J_w,i_* is the water flux in the *i*th stage, *c_p,i_* is the permeate concentration in the *i*th stage, and *S_m,i_* is the membrane area in the *i*th stage. The simulation takes into account the dilution and concentration within the module. For example, the concentration effect on the feed side of a module is calculated using Equations (11) and (12). The dilution effect on the exhaust side of a module is calculated using Equations (13) and (14). The average flux in each stage is also calculated by taking into account the dilution and concentration effects.

Here, the recovery of the *i*th stage (*RR_i_*) is defined as

(15) $$RR_{i}=\frac{Q_{f1,i}-Q_{f2,i}}{Q_{f1,i}}=\frac{J_{w,i}S_{m,i}}{Q_{f1,i}}$$

A part of the salt is separated from the total inflow to the concentrated feed (or the brine) for the *i*th stage. To simplify the simulation, the *RR_i_* values are assumed to be the same in all stages. This implies that the membrane area in each stage (*S_m,i_*) is a function of *RR_i_* and *J_w,i_*.

(16) $$S_{m,}{}_{i}=\frac{RR_{i}Q_{f1,i}}{J_{w,i}}$$

Since *RR_i_* is fixed, *J_w,i_* is explicitly calculated in each stage. Then, the *S_m,i_* is estimated using the above equation. In a similar way, the separation efficiency for salts can be calculated, which is quantified by the ratio of the salt in the brine to the total inflow (*SR_i_*):

(17) $$SR_{i}=\frac{c_{f2,i}Q_{f2,i}}{c_{T,i}Q_{f1,i}}=\frac{c_{f2,i}Q_{f2,i}}{c_{f2,i}Q_{f2,i}+c_{d2,i}Q_{d2,i}}$$

As shown in Figure 2, the total inflow (*Q_T,i_*) is the sum of the concentrated feed flow from the previous stage (*Q_f_*_2,*i*−1_) and the diluted draw from the next stage (*Q_f_*_2,*i*−1_). This leads to the following equations:

(18) $$Q_{T,i}=Q_{f2,i-1}+Q_{d2,i+1}$$

(19) $$c_{t,i}Q_{T,i}=c_{f2,i-1}Q_{f2,i-1}+c_{d2,i+1}Q_{d2,i+1}$$

where *Q_f_*_2,*i*−1_ is the flow rate of the concentrated solution from the feed side of the membrane in the (*i* − 1)th stage, *Q_d2,i+1_* is the flow rate of the diluted solution from the draw side of the membrane in the (*i* + 1)th stage, *c_f_*_2*,i−1*_ is the concentration of the concentrated solution from the feed side of the membrane in the (*i* − 1)th stage, and *c_d2,i_*_+1_ is the concentration of the diluted solution from the draw side of the membrane in the (*i* + 1)th stage.

The rejection of the salt in each stage is defined as Equation (20) and can be calculated using Equation (21):

(20) $$R_{i}=1-\frac{c_{p,i}}{{\bar{c}}_{f,i}}$$

(21) $$c_{p,i}=\frac{J_{s,i}}{J_{w,i}}$$

where ${\bar{c}}_{f,i}$ is the average concentration of the feed in the *i*th stage, and *J_s,i_* is the salt flux in the *i*th stage.

## 3. Materials and Methods

### 3.1. Feed Solution

The feed and draw solutions used in this study were a NaCl solution simulating the seawater reverse osmosis (SWRO) brine. The concentration of these solutions varied from 30 g/L to 130 g/L depending on the purpose of the experiment. There was no additional foulant in the draw solution because the possibility of membrane fouling was not considered in this study.

Due to the lack of other ions and dissolved organics, the synthetic brine has different properties than the real SWRO brine, and one of the most important differences is the fouling propensity. Since the focus of this study was to develop and apply a theoretical model for OARO under non-fouling conditions, the use of the current synthetic brine can be justified. In addition, a recent study on OARO proposed the use of nanofiltration (NF) as a pretreatment for OARO [27]. In such cases, the OARO feed in such a system may mainly contain NaCl. Of course, a synthetic brine containing not only NaCl but also other ions and organics should be used to study fouling and scaling, which will be accomplished in future work.

### 3.2. Membranes

The membrane used in the experiments was a commercial-grade Thin-Film Composite (TFC) membrane (CSM-PRO-4, Toray Chemical Korea, Republic of Korea), which was originally developed for pressure-retarded osmosis (PRO). The membranes required for OARO experiments must be pressure-resistant (up to at least 20 bar) and capable of FO operation, and this PRO membrane fulfills these conditions. The membrane comprises an active layer made of polyamide (PA) and a woven polymeric support layer. The support layer of the membrane has relatively large pores, the size of which varies from 10~20 μm. The water permeability (*A*), salt permeability (*B*), and structural parameter (*S*) of the membrane provided by the manufacturer were 2.85 L/m^2^-hr-bar, 0.465 L/m^2^-hr, and 480 μm, respectively [31]. The water and salt permeabilities were also confirmed by laboratory-scale RO tests. There are some differences between these membrane properties and those in the literature. For example, a previous study on OARO simulation assumed that *A*, *B*, and *S* values are 1.5 L/m^2^-hr-bar, 0.045 L/m^2^-hr, and 1200 μm. This is because the membrane in this study was originally designed for PRO process, which requires membrane with higher permeability of both water and salt than conventional RO membranes. Prior to testing, both sides of the membrane samples were thoroughly rinsed and stored in deionized (DI) water at 4 °C.

### 3.3. Experimental Setup

**Figure 3:**
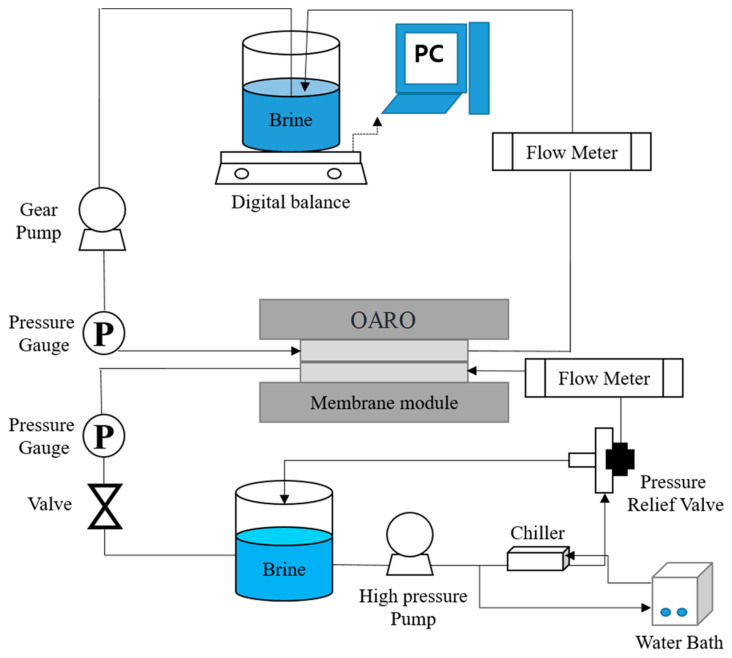
Schematic diagram of lab-scale experimental setup for OARO.

A laboratory-scale experimental setup for OARO operation was used (Figure 3), which was modified from our experimental setup for PRO in previous studies [32,33]. As shown in Figure 3, it included a feed water tank, a draw solution tank, a low-pressure pump for the draw solution, a high-pressure pump for the feed water, a plate-and-frame OARO membrane module, an electronic balance for flux measurement, pressure sensors, a temperature control system, and flow meters. The OARO module was designed to have two channels, and the experiment was conducted in a countercurrent flow. The effective membrane area was 0.014 m^2^ (95.01 mm × 145.58 mm). Both feed and draw flow rates were 0.5 L/min. Using Equations (4) and (5), the *k_D_* and *k_f_* were calculated as 0.1 m^2^-hr/L and 7.55 × 10^3^ L/m^2^-hr, respectively. Since *k_f_* is larger than 1/*k_D_*, it can be concluded that the internal concentration polarization is more important than the external concentration polarization [30]. The volumes of the feed and draw solutions were both 2 L. The feed solution was recycled, which may affect the solute concentration during the experiment. Nevertheless, these changes were negligible because the membrane area (0.014 m^2^) was small compared to the initial feed volume (2 L).

The details on the lab-scale experiments are summarized in Table 1. Since the main purpose of the experimental investigations was to verify the theoretical model, their operating conditions were determined to serve this purpose. The concentration of the feed and draw solutions was varied from 50 g/L to 130 g/L. The applied pressure was adjusted to 5, 10, 15, and 20 bar, respectively. The effect of the operating conditions on the performance of the OARO was investigated using the theoretical model, which was verified using the experimental data. Since the model can predict the performance of OARO systems, it can help improve their robustness.

### 3.4. Simulation for Full-Scale OARO System

A full-scale OARO system was simulated to quantify its potential for MLD. First, the flux models (from Equations (2)–(5) were verified using the results from the lab-scale experiments. Then, these model equations were combined with the mass balance equations listed by Equations (6)–(21). The full-scale OARO process was assumed to have four OARO stages and one seawater RO (SWRO) stage. The salinity of the feed to the 1st OARO was fixed at 50 g/L. The feed flow rate was set to 1.0 m^3^/hr. The effect of operating conditions on the performance of the full-scale OARO processes was investigated as a function of Δ*P*, *RR_i_*, and *FR_i_*. To reduce the cases for the simulation, the following assumptions were made:

- *RR_i_* and *FR_i_* are the same in all OARO stages.
- The concentration of the final brine should be close to 130 g/L. If it cannot be obtained, the maximum attainable concentration is presented instead.
- The concentration of the diluted draw stream should be close to 30 g/L, which is similar to the concentration of the SWRO.
- Based on these assumptions, 9 total cases for the simulation were prepared. Table 2 summarizes the simulation conditions for the full-scale OARO system. For each case, the model equations were simultaneously solved using MATLAB. After the simulation, the flux, overall *SR*, and final *RR* were analyzed and compared among the cases.

## 4. Results

### 4.1. Lab-Scale Experiments: Effects of Pressure and Concentrations

**Figure 4:**
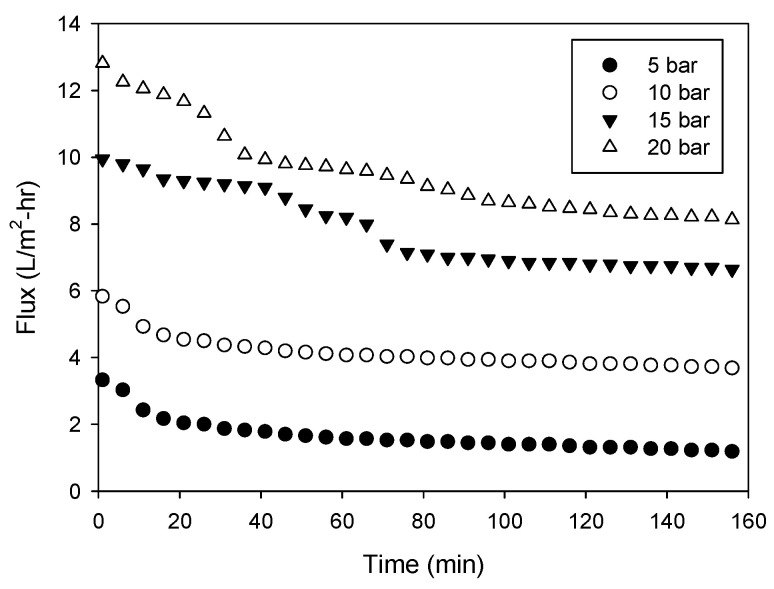
Changes in flux with time for different applied pressures in lab-scale OARO (conditions—feed: 50 g/L NaCl; draw: 50 g/L NaCl).

A series of laboratory scale experiments were conducted to measure the flux and concentrations of the feed and draw solutions under various conditions. Figure 4 shows the flux versus time under different pressure conditions. The initial concentrations of the feed and draw solutions were both 50 g/L, indicating that there is no difference in apparent osmotic pressure between the two solutions. The applied pressure (or transmembrane pressure) was adjusted from 5 bar to 20 bar. As the pressure increased, the initial flux increased due to a higher driving force. For example, the initial flux at 5 bar was only 3.32 L/m^2^-hr, while that at 20 bar was 12.8 L/m^2^-hr, which is approximately 3.85 times higher. It should be noted that the feed solution has an osmotic pressure of approximately 44.6 bar. No water can pass through the membrane when the feed concentration is low, which is the case in conventional RO processes.

As the experiment continued, the flux decreased with time. These results were not caused by membrane fouling because only NaCl was used to prepare the feed solution. Instead, they were due to the dilution effect. Initially, the osmotic pressure difference between the feed and draw solutions was negligible. However, it increased over time as the feed was concentrated and the draw was diluted. Table 3 shows the initial and final concentrations of the feed and draw solutions in these OARO runs. At the end of the runs, the final concentration difference ranged from 1.5 g/L to 11.3 g/L, corresponding to the osmotic pressure differences ranging from 1.2 bar to 8.6 bar.

**Figure 5:**
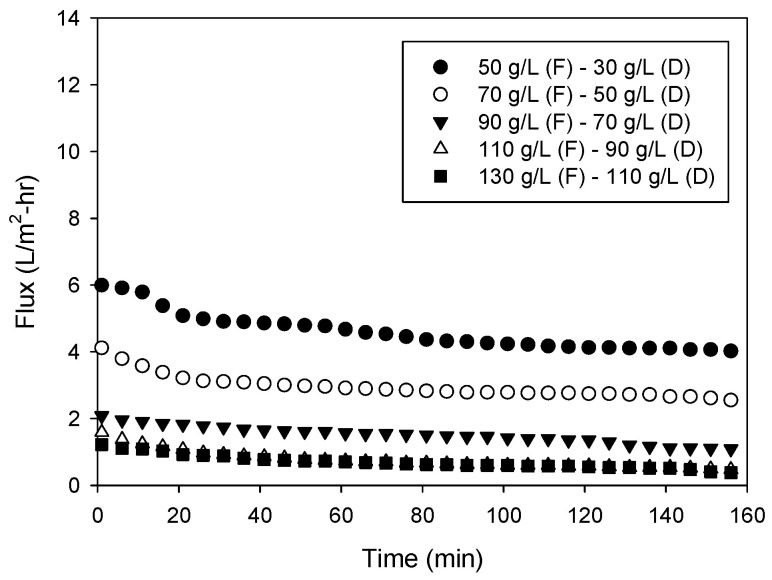
Changes in flux with time for different concentrations of feed and draw solutions in lab-scale OARO (conditions—applied pressure: 20 bar).

Figure 5 shows the flux profiles for different combinations of the feed and draw solutions. In all cases, the concentration difference was fixed at 20.0 g/L. Nevertheless, the initial and final flux values were quite different. For instance, the flux was initially 6.0 L/m^2^-hr and decreased to 4 L/m^2^-hr when the feed and draw concentrations were 50.0 g/L and 30.0 g/L, respectively. On the other hand, the initial and final fluxes were 0.51 L/m^2^-hr and 0.34 L/m^2^-hr, respectively, when the feed concentration was 130 g/L and the draw concentration was 110 g/L. The flux values were also different in the other cases. These results are attributed to the concentration polarization in OARO. Similar to FO, OARO suffers from internal and external concentration polarization [24]. As shown in Equation (2), the flux may be different even with the same concentration difference between the feed and draw due to the terms related to the concentration polarization, $e^{-J_{w}k_{D}}$ and $e^{J_{w}/k_{F}}$. From the results, it can be concluded that the flux becomes lower as the feed concentration becomes higher, even if the concentration difference is constant.

The initial and final concentrations of the feed and draw solutions in the previous cases are summarized in Table 4. The initial feed solutions had osmotic pressures ranging from 44.6 bar to 104.3 bar, which cannot be treated by conventional RO with an applied pressure of 20 bar. Although the tests were conducted in a laboratory-scale system in a short period of time, it was experimentally confirmed that the feed solution with high osmotic pressure can be further concentrated by OARO with relatively low pressure. This implies that OARO could be used to reduce the volume of SWRO brine, even at an affordable pressure.

### 4.2. Model Verification

**Figure 6:**
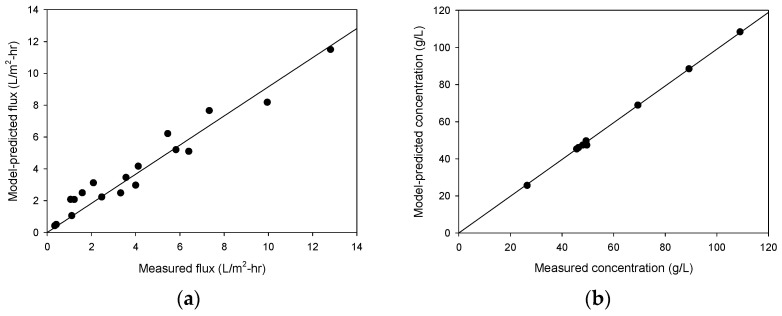
Model verification. (**a**) Flux and (**b**) concentration of draw solution.

Using the model’s equations (from Equations (2)–(5), the flux and draw concentration in OARO are calculated to predict the experimental results using the membrane parameters provided by the membrane manufacturers. In Figure 6, the results from the theoretical model are compared with the actual experimental data for the flux and draw concentration after the runs. These show a reasonable agreement between the two, suggesting that the model accurately represents the phenomena under study. The R^2^ values for the flux and draw concentration were 0.944 and 0.999, respectively. This agreement is not perfect, especially for the flux data, but is close enough to validate the predictions of the model. It also provides confidence in the applicability to the particular experiment or system being analyzed.

Although the theoretical model was experimentally validated using the experimental data, it should be noted that there are still limitations. The laboratory-scale experiments were performed on membrane coupons within a short time (160 min). Since the membrane elements used in a full-scale process may not have different properties from the membrane coupons, there may be some uncertainty in the full-scale simulation using this model. Moreover, the model does not consider the effects of membrane fouling and scaling. In addition, the model assumes that the membrane properties are constant over time. Therefore, it is important to understand that the model may have potential discrepancies in some situations.

### 4.3. Full-Scale Simulation

**Figure 7:**
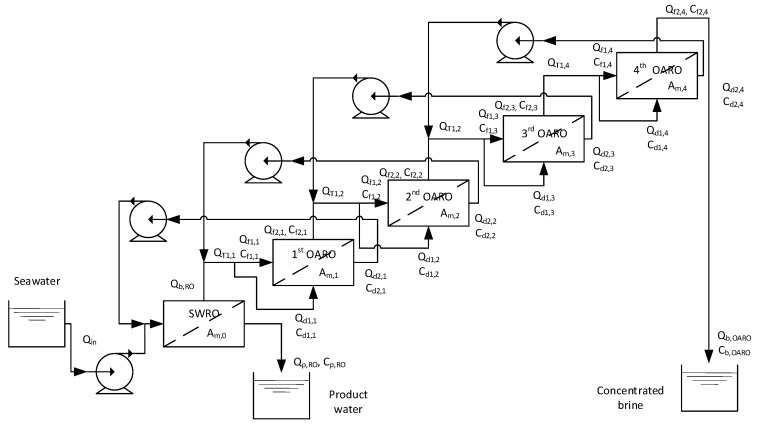
Full-scale OARO system consisting of a single-stage SWRO and four-stage OARO.

Using the flux models (from Equations (2)–(5)) in combination with the mass balance equations (from Equations (6)–(21)), a full-scale OARO system was simulated to check its feasibility and understand the effect of the operating variables. As mentioned above, a four-stage OARO system was assumed to be integrated into an SWRO process. The schematics of this full-scale system is illustrated in Figure 7. Seawater with a salinity of 30 g/L is fed into the SWRO process. The SWRO brine is then used as an inflow to the first stage of the OARO system. In each stage, the diluted draw from the next stage (*Q_d_*_2,*i+1*_) is returned to the current stage (*Q_f_*_2,*i−1*_) and mixed with the influent from the previous stage. The sum of these two flows becomes the total inflow (*Q_T,i_*), which is divided into the feed (*Q_f_*_1,*i*_) and the draw (*Q_d_*_1,1_). The concentrated feed in the last stage (*Q_f_*_2,4_) becomes the final brine (*Q_b,OARO_*), and the diluted draw in the first stage (*Q_d_*_2,1_) is returned to the SWRO process. The mass balance equations describe these relationships.

**Figure 8:**
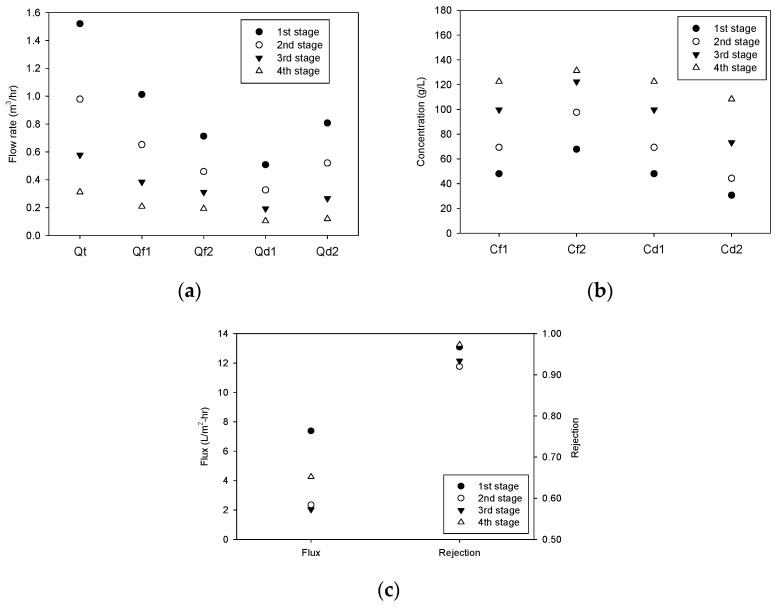
Simulation results for full-scale OARO system. (**a**) Flow rate, (**b**) concentration, and (**c**) flux and rejection.

The cases for the simulation are summarized in Table 2. Among them, a representative case (the second case in Table 2) was selected, and the simulation was carried out. In this case, ∆*P*, *RR_i_*, and *FR_i_* are 25 bar, 0.255, and 0.667, respectively. Figure 8a shows how the flow rates of the feed and the draw streams change in different stages. The flow rates decrease from the first stage to the fourth stage. The difference between *Q_f_*_1,*i*_ and *Q_f_*_2,*i*_ corresponds to the permeate flow rate (*Q_p,i_*), which is the same as the difference between *Q_d_*_2,*i*_ and *Q_d_*_1,*i*_. The OARO feed flow rate (*Q_b,RO_*) is 1.0 m^3^/hr, and the final brine (*Q_b,OARO_*) is reduced to 0.194 m^3^/hr, which corresponds to approximately 81% of the recovery. Please note that the applied pressure for OARO is only 25 bar, which is lower than the osmotic pressure of the OARO feed (44.6 bar).

**Figure 9:**
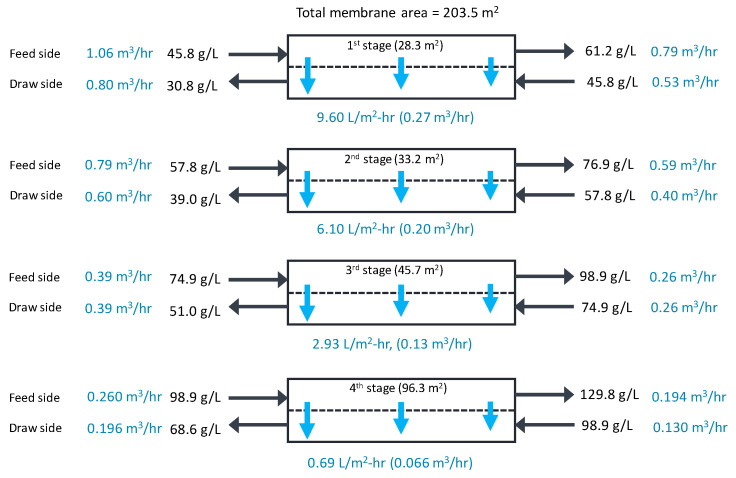
Simulation results for flow rates and concentrations in each stage of the full-scale OARO system.

As shown in Figure 8b, the salt concentrations increase from the first stage to the fourth stage. The initial feed concentration is 50.0 g/L, and the outflow concentrations in the first, second, third, and fourth stages are 61.1 g/L, 76.9 g/L, 98.9 g/L, and 129.8 g/L, respectively. The difference in the average concentrations between the feed and draw streams in these stages ranges from 15.2 g/L to 30.6 g/L. Due to different concentrations, the flux and rejection in the stages are different, as presented in Figure 8c. The flux ranges from 9.60 L/m-hr (first stage) to 0.69 L/m^2^-hr (fourth stage), and the rejection ranges from 0.974 (first stage) to 0.910 (fourth stage). These results are also shown in Figure 9.

The final brine concentration after the four-stage OARO system is approximately 130 g/L in this case. According to the literature, the final brine concentrations from different brine concentration technologies ranged from 169 g/L to 250 g/L [27]. A combination of NF–SWRO–OARO resulted in a final concentration of 169 g/L. A combination of RO with electrodialysis (ED) was reported to achieve up to 244 g/L. Two-stage MVC can also accomplish a final concentration of 250 g/L. The current OARO system can also increase the final concentration by adjusting the operating variables. This is discussed in the next section.

In this simulation, the ratio of the feed to the permeate flux is fixed (0.667) in all stages. Accordingly, the membrane area required in a stage should be larger when the flux is lower. Figure 9 shows the calculated membrane areas for the stages. Although the feed flow is the highest in the first stage (1.06 m^3^/h), the required membrane area is the smallest (28.2 m^2^). Conversely, the smallest feed flow in the fourth stage (0.260 m^3^/hr) requires the largest membrane area (96.3 m^2^). The capital cost of the membrane system generally increases as the membrane area requirement increases, which is inversely proportional to the flow. Therefore, it is important to understand how operating variables affect the performance and cost of this system.

Although the main focus of this study is on the process simulation under non-fouling conditions, the effect of membrane fouling on process efficiency is briefly considered using the model. Assuming that membrane fouling reduces the A value by 20%, the model calculates that the flux in the first stage decreases from 9.60 L/m^2^-hr to 8.90 L/m^2^-hr. Similar results can be obtained in the other stages. To keep the mass balance of the process, it is necessary to increase the flux by increasing the applied pressure. In the above case, the flux in the first stage can be restored to 9.60 L/m^2^-hr if the applied pressure increases by 0.9 bar. This example highlights that understanding and controlling the operating variables are important in OARO systems. Accordingly, an in-depth analysis of the effect of the operation variables are performed in the next section.

### 4.4. Effect of the Operating Variables

Table 5 shows the flow rates, concentrations, and membrane performances of OARO systems as a function of the applied pressure, adjusted from 20 bar to 25 bar (cases 1, 2, and 3 in Table 2). The *RR_i_* and *FR_i_* are constant in this simulation. When the applied pressure is 20 bar, the final brine concentration (*C_f_*_2,4_) is only 96.4 g/L. This is because the applied pressure is not sufficient to overcome the effective osmotic pressure difference between the feed and draw in the third and fourth stages. In fact, the calculated flux in these stages is zero due to an insufficient driving force. On the other hand, the final brine concentrations reach approximately 130 g/L at 25 bar and 30 bar. The average flux values are 3.31 L/m^2^-hr at 25 bar and 7.04 L/m^2^-hr at 30 bar. However, the separation ratio remains almost constant. This means that the relative quantity of salts in the final brine is not changed by adjusting the applied pressure.

In Table 6, the applied pressure is fixed at 25 bar, and the stage recovery (*RR_i_*) is varied from 0.21 to 0.255 (case 2, 4, 5, and 6). The feed ratio (*FR_i_*) is the same as the previous cases. As *RR_i_* increases, the final brine concentration (*C_f_*_2,4_) increases. At the same time, the average flux decreases with *RR_i_*. This is because the difference between the feed and discharge concentrations in a stage increases with *RR_i_*. The overall recovery (*RR_T_*) increases with increasing *RR_i_*, indicating the amount of the final brine is reduced. In contrast, the separation ratio is not significantly affected by *RR_i_*.

The effect of the feed ratio (*FR_i_*) on the flow rates, concentrations, and flux is shown in Table 7. When *FR_i_* varies from 0.583 to 0.833, the final brine concentration (*C_f2,4_*) decreases from 152.7 g/L to 93.0 g/L. The recovery is also affected, which is reduced from 0.89 to 0.57. This implies that as *FR_i_* increases, a greater amount of brine is produced. It is interesting to note that SR increases with increasing *FR_i_*. This is attributed to the decreased concentration of the diluted draw stream (*C_d_*_2,1_). Since a smaller amount of the salt exits in the diluted draw, the amount of the salt in the final brine increases, thereby increasing the *SR*. The dependence of the flux on *FR_i_* is not clear because the flux increases and decreases with *FR_i_*.

Based on these simulation results, the effect of the operating variables is summarized as follows:

- As ∆*P* increases, the final brine concentration increases along with the flux.
- An increase in *RR_i_* results in an increase in the final brine concentration, a decrease in the flux, and an increase in the total recovery.
- With an increase in *FR_i_*, the final brine concentration, as well as the total recovery, decreases. In contrast, SR increases with *FR_i_*.

To further investigate the effect of the operating variables, a preliminary sensitivity analysis is performed. From the simulation results shown in Table 5, Table 6 and Table 7, a data set is selected to calculate the variations in operating variables such as applied pressure, stage recovery, and feed ratio. The relative changes in the flux and recovery are then evaluated. These results are used to estimate the sensitivity of the flux or recovery for each operating variable. The results are summarized in Table 8. It is found that the flux and recovery are most sensitive to the stage recovery and feed ratio, respectively. In other words, the stage recovery has the most influence on the flux, while the feed ratio is the most critical in determining the recovery.

### 4.5. Energy Consumption

To evaluate the energy efficiency of OARO, the theoretical power consumption is calculated using the following equations [23]. As shown in Figure 7, the SWRO brine and the diluted draw returning back to the OARO stages should be pressurized up to their applied pressures (Δ*P_f1,i_*), resulting in the total power consumption during the OARO stages (*EC_OARO_*).

(22) $$EC_{OARO}=Q_{b,RO}\Delta P_{f1,1}+\sum_{i=2}^{N}Q_{d2,i}\Delta P_{f1,i}$$

where *Q_b,RO_* is the flow rate of the brine in the previous SWRO stage. Then, the specific energy consumption (*SEC_OARO_*) to dilute the brine to a desired level (i.e., similar to the concentration of the seawater) is calculated by

(23) $$SEC_{OARO}=\frac{EC_{OARO}}{\eta_{pump}Q_{d2,1}}$$

where *η_Pump_* is the efficiency of the high-pressure pump in OARO stages.

There are several different ways to define the SEC of OARO: (1) based on the feed solution volume [34], (2) based on the product volume [34], and (3) based on the permeate volume [31]. Although the definition in the third case is often used, it is the definition for the SEC of the OARO-RO hybrid system, which cannot be directly applied to calculate the SEC for OARO alone. To calculate the SEC, Equation (23) uses *Q*_*d*2,1_, which is the “permeate” of the OARO system. As shown in Figure 7, the SWRO brine is fed to the first OARO stage and is concentrated in stages. At the end of the system, the final concentrate stream is obtained, corresponding to *Q*_*f*2,4_. In contrast, the final permeate stream leaving the OARO system is *Q*_*d*2,1_, which returns to the feed of the SWRO. Accordingly, *Q*_*d*2,1_ was used as the basis for the SEC calculation. Although this is not a standard definition for the SEC, it can be used for relative comparison purposes only.

As an example, the SEC was calculated for case 2 in Table 2, where Δ*P*, *RR_i_*, and *FR_i_* are 25 bar, 0.255, and 0.667, respectively. The *η_Pump_* is assumed to be 0.8, and the pressure drops in the stages are neglected. Based on these assumptions, the calculated *EC_OARO_* is 1.54 kW. Accordingly, *SEC_OARO_* is estimated to 2.38 kWh/m^3^. It should be noted that this calculation does not consider the power required to treat the diluted draw (*Q*_*d*2,1_) in the SWRO stage. If this is included, the actual SEC (or *SEC_total_*) would be much higher. Moreover, the capital cost of the OARO system may be high due to its low flux (i.e., 3.31 L/m^2^-hr in case 2).

Nevertheless, the SEC for OARO is still much lower than that for other brine concentration processes such as MED and MVC. For MED–MVC, the SEC lies in the range between 7 and 12 kWh/m^3^ [35]. For MED–TVC (thermal vapor compression), the SEC is higher, ranging between 14 and 22 kWh/m^3^ [36]. Although it may not be a fair comparison, this still suggests that OARO has potential for reducing the SWRO brine in an affordable manner. A previous work on OARO also reported that the SEC for an OARO process (6 kWh/m^3^) is much lower than that of MVC (>20 kWh/m^3^) [27]. The challenges associated with low flux and productivity may be addressed in the future through the development of novel OARO membranes [28].

Accordingly, it appears that a full-scale OARO system has potential for large-scale implementation. Compared with the conventional thermal techniques such as MED and MVC, the energy consumption of OARO is much less. Moreover, the capital cost of OARO may be also lower than that of MED and MVC, which require the use of expensive materials for the construction of pipes and evaporators [21]. A recent study on the techno-economic analysis results reported that a large-scale brine concentration system including OARO has economic feasibility [27].

## 5. Conclusions

This paper provides an exploration of OARO in laboratory-scale experiments and a theoretical investigation using a full-scale process simulation model. The feasibility of OARO for minimum liquid discharge (MLD) is investigated using a polyamide membrane, and the effect of key operating variables on OARO performance is elucidated to provide insight into the process design and optimization. The following conclusions can be drawn:

- The concept of the OARO process is experimentally verified. Although the applied pressure is much lower than the osmotic pressure of the feed solution, the OARO could further concentrate it with the help of the draw solution.
- The effect of the pressure (hydraulic driving force) and the concentration (osmotic driving force) on the OARO performance is experimentally investigated. The flux ranges from 0.34 L/m^2^-hr to 12.8 L/m^2^-hr depending on the conditions. Although the difference between the feed and draw concentrations is the same, the flux is different with a different initial feed concentration, which is attributed to the concentration polarization effect.
- The model equations for predicting the flux and salt concentration are verified with experimental results at the laboratory scale. The model agrees well with the experimental flux and concentration, resulting in an *R*^2^ of 0.944 and 0.999, respectively.
- The simulation results indicate that a four-stage OARO system can treat the SWRO brine and increase the concentration from 50 g/L to 130 g/L by applying a transmembrane pressure of only 25 bar. This can be achieved by combining the hydraulic pressure and the osmotic pressure across the membrane. The recovery (or volume reduction in the SWRO brine) ranges from 0.57 to 0.89.

This study demonstrates the potential of a full-scale OARO system as a brine concentration technology. However, it should be noted that scaling up from a lab-scale system to a full-scale industrial process is challenging. Although the lab-scale results are promising, the availability of full-scale membrane modules is key to scaling up. In addition, the hydrodynamic conditions between lab-scale and full-scale systems lead to unexpected results after scaling up. A techno-economic study is also essential prior to scaling up OARO systems. In conclusion, further work is recommended for the full-scale implementation of OARO systems.

## Figures and Tables

**Table 1:** Summary of parameters for lab-scale experiments.

Parameter	Value
Feed concentration	50~130 g/L (NaCl)
Feed flow rate	0.5 L/min
Draw flow rate	0.5 L/min
Flow direction	Counter-current
Applied pressure	5, 10, 15, and 20 bar
Initial feed volume	2 L
Initial draw volume	2 L
Operation time	160 min
Membrane water permeability (*A*)	2.85 L/m^2^-hr-bar
Membrane salt permeability (*B*)	0.465 L/m^2^-hr
Structural parameter (*S*)	480 μm
Effective membrane area (*S_m_*)	0.014 m^2^

**Table 2:** Operating conditions for full-scale OARO system.

Case	Feed Conditions		Operating Conditions		
	Flow Rate (m^3^/hr)	Concentration (g/L)	Δ*P* (bar)	*RR_i_*	*FR_i_*
1	1.0	50.0	20	0.255	0.667
2	1.0	50.0	25	0.255	0.667
3	1.0	50.0	30	0.255	0.667
4	1.0	50.0	25	0.210	0.667
5	1.0	50.0	25	0.225	0.667
6	1.0	50.0	25	0.240	0.667
7	1.0	50.0	25	0.255	0.583
8	1.0	50.0	25	0.255	0.75
9	1.0	50.0	25	0.255	0.833

**Table 3:** Effect of applied pressure on concentrations of feed and draw in lab-scale OARO.

Applied Pressure (bar)	Initial		Final	
	Feed (g/L)	Draw (g/L)	Feed (g/L)	Draw (g/L)
5	50.0	50.0	51.5	49.6
10	50.0	50.0	53.2	47.4
15	50.0	50.0	55.7	46.0
20	50.0	50.0	56.6	45.3

**Table 4:** Effect of initial concentrations of feed and draw solutions in lab-scale OARO.

Applied Pressure (bar)	Initial		Final	
	Feed (g/L)	Draw (g/L)	Feed (g/L)	Draw (g/L)
20	50.0	30.0	55.9	25.6
20	70.0	50.0	74.3	47.4
20	90.0	70.0	92.2	68.9
20	110.0	90.0	111.6	88.5
20	130.0	110.0	130.8	108.4

**Table 5:** Simulation results for full-scale OARO system: effect of applied pressure.

Applied Pressure (bar)	Inflow		OARO Concentrate		OARO Product (Return to SWRO)		Membrane Performance		
	Stage Recovery	Feed Ratio	Flow Rate (L/min)	Concentration (g/L)	Flow Rate (L/min)	Concentration (g/L)	Flux (L/m^2^-hr)	Separation Ratio	Recovery
	*RR_i_*	*FR_i_*	*Q_f_*_2,4_	*C_f_*_2,4_	*Q_d_*_2,1_	*C_d_*_2,1_	*J_v_*	*SR*	*RR_T_*
20	0.255	0.667	0.26	96.4	0.74	33.6	1.88	0.505	0.74
25	0.255	0.667	0.19	129.8	0.81	30.8	3.31	0.503	0.81
30	0.255	0.667	0.19	130.5	0.81	30.6	7.04	0.506	0.81

**Table 6:** Simulation results for full-scale OARO system: effect of stage recovery.

Stage Recovery (*RR_i_*)	Operating Conditions		OARO Concentrate		OARO Product (Return to SWRO)		Membrane Performance		
	Applied Pressure (bar)	Feed Ratio	Flow Rate (m^3^/hr)	Concentration (g/L)	Flow Rate (m^3^/hr)	Concentration (g/L)	Flux (L/m^2^-hr)	Separation Ratio	Recovery
	Δ*P*	*FR_i_*	*Q*_*f*2,4_	*C*_*f*2,4_	*Q*_*d*2,1_	*C*_*d*2,1_	*J_v_*	*SR*	*RR_T_*
0.210	25	0.667	0.24	104.1	0.76	32.6	7.11	0.508	0.76
0.225	25	0.667	0.23	111.5	0.77	32.0	6.19	0.505	0.77
0.240	25	0.667	0.21	120.0	0.79	31.4	5.05	0.504	0.79
0.255	25	0.667	0.19	129.8	0.81	30.8	3.31	0.503	0.81

**Table 7:** Simulation results for full-scale OARO system: effect of feed ratio.

Feed Ratio (*FR_i_*)	Operating Conditions		OARO Concentrate		OARO Product (Return to SWRO)		Membrane Performance		
	Applied Pressure (bar)	Stage Recovery	Flow Rate (m^3^/hr)	Concentration (g/L)	Flow Rate (m^3^/hr)	Concentration (g/L)	Flux (L/m^2^-hr)	Separation Ratio	Recovery
	Δ*P*	*RR_i_*	*Q*_*f*2,4_	*C*_*f*2,4_	*Q*_*d*2,1_	*C*_*d*2,1_	*J_v_*	*SR*	*RR_T_*
0.583	25	0.255	0.11	152.7	0.89	37.2	3.23	0.337	0.89
0.667	25	0.255	0.19	129.8	0.81	30.8	3.31	0.503	0.81
0.75	25	0.255	0.30	108.5	0.70	24.5	4.96	0.659	0.70
0.833	25	0.255	0.43	93.0	0.57	18.2	3.02	0.790	0.57

**Table 8:** Sensitivity analysis for full-scale OARO system: relative impact of operating variables on flux and recovery.

Operating Variable	Variation in Operating Variable (%)	Change in Flux (%)	Change in Recovery (%)	Sensitivity of Flux	Sensitivity of Recovery
*x*	Δ*x*/*x*	Δ*J_v_*/*J_v_*	Δ*RR_T_*/*RR_T_*	(Δ*J_v_*/*J_v_*)/(Δ*x*/*x*)	(Δ*RR_T_*/*RR_T_*)/(Δ*x*/*x*)
Applied pressure	−20.0	−43.0	−8.6	+2.16	+0.43
Stage recovery	−17.6	+114.8	−6.2	−6.5	+0.35
Feed ratio	−12.6	−2.4	+9.8	+0.19	−0.78

## Data Availability

Not applicable.

## Nomenclature

*A*	water permeability of the membrane (L/m^2^-hr-bar)
*B*	salt permeability of the membrane (L/m^2^-hr)
*c_F_*	salt concentration of the feed solution (mole/L)
*c_D_*	salt concentration of the draw solution (mole/L)
*c_T,i_*	concentration of the total inflow in the *i*th stage (g/L)
*c*_*f*1,*i*_	concentration of the solution supplied to the feed side of the membrane in the *i*th stage (g/L)
*c*_*d*1,*I*_	concentration of the solution supplied to the draw side of the membrane in the *i*th stage (g/L)
*c*_*f*2,*i*_	concentration of the concentrated solution from the feed side of the membrane in the *i*th stage (g/L)
*c*_*d*2,*i*_	concentration of the diluted solution from the draw side of the membrane in the *i*th stage (g/L)
*c_p,i_*	permeate concentration in the *i*th stage
*D*	diffusion coefficient of the solute (m^2^/s)
*FR_i_*	ratio of the feed to the total inflow
*J_w_*	water flux (L/m^2^-hr)
*J_s_*	salt flux (mole/m^2^-hr)
*J_w,i_*	water flux in the *i*th stage (L/m^2^-hr)
*J_s,i_*	salt flux in the *i*th stage (mole/m^2^-hr)
*k_F_*	mass-transfer coefficient related to external concentration polarization (L/m^2^-hr)
*k_D_*	mass-transfer resistance related to internal concentration polarization (m^2^-hr/L)
*L_h_*	length of the membrane channel (m)
*P_H_*	pressure on the high-salinity solution side
*P_L_*	pressure on the low-salinity solution side
*DP*	transmembrane pressure between the high-salinity and low-salinity solutions
*Q_T,i_*	flow rate of the total inflow in the *i*th stage (m^3^/hr)
*Q*_*f*1,*i*_	flow rate of the solution supplied to the feed side of the membrane in the *i*th stage (m^3^/hr)
*Q*_*d*1,*i*_	flow rate of the solution supplied to the draw side of the membrane in the *i*th stage (m^3^/hr)
*Q*_*f*2,*i*_	flow rate of the concentrated solution from the feed side of the membrane in the *i*th stage (m^3^/hr)
*Q*_*d*2,*i*_	flow rate of the diluted solution from the draw side of the membrane in the *i*th stage (m^3^/hr)
*S*	structural parameter of the membrane (m)
*Sh*	Sherwood number
*S_m,i_*	membrane area in the *i*th stage
*SR_i_*	ratio of the salt in the brine to the total inflow
*ν*	kinematic viscosity (m^2^/s)
*Dπ*	transmembrane osmotic pressure between the high-salinity and low-salinity solutions
*π_F_*	osmotic pressure of the feed solution (bar)
*π_D_*	osmotic pressure of the draw solution (bar)
*d_h_*	hydraulic diameter (m)
*u_h_*	crossflow velocity (m/s)
